# Machine learning algorithms reveal potential miRNAs biomarkers in gastric cancer

**DOI:** 10.1038/s41598-023-32332-x

**Published:** 2023-04-15

**Authors:** Hanieh Azari, Elham Nazari, Reza Mohit, Alireza Asadnia, Mina Maftooh, Mohammadreza Nassiri, Seyed Mahdi Hassanian, Majid Ghayour-Mobarhan, Soodabeh Shahidsales, Majid Khazaei, Gordon A. Ferns, Amir Avan

**Affiliations:** 1 Metabolic Syndrome Research Center, Mashhad University of Medical Sciences, Mashhad, Iran; 2 Basic Sciences Research Institute, Mashhad University of Medical Sciences, Mashhad, Iran; 3 Medical Genetics Research Center, Mashhad University of Medical Sciences, Mashhad, Iran; 4 Department of Anesthesia, Bushehr University of Medical Sciences, Bushehr, Iran; 5 Recombinant Proteins Research Group, The Research Institute of Biotechnology, Ferdowsi University of Mashhad, Mashhad, Iran; 6 Cancer Research Center, Mashhad University of Medical Sciences, Mashhad, Iran; 7 Division of Medical Education, Brighton and Sussex Medical School, Falmer, Brighton, Sussex, BN1 9PH UK; 8 Faculty of Health, School of Biomedical Sciences, Queensland University of Technology, Brisbane, Australia; 9 College of Medicine, University of Warith Al-Anbiyaa, Karbala, Iraq, College of Medicine, University of Warith Al-Anbiyaa, karbala, Iraq

**Keywords:** Cancer, Computational biology and bioinformatics, Genetics, Biomarkers

## Abstract

Gastric cancer is the high mortality rate cancers globally, and the current survival rate is 30% even with the use of combination therapies. Recently, mounting evidence indicates the potential role of miRNAs in the diagnosis and assessing the prognosis of cancers. In the state-of-art research in cancer, machine-learning (ML) has gained increasing attention to find clinically useful biomarkers. The present study aimed to identify potential diagnostic and prognostic miRNAs in GC with the application of ML. Using the TCGA database and ML algorithms such as Support Vector Machine (SVM), Random Forest, k-NN, etc., a panel of 29 was obtained. Among the ML algorithms, SVM was chosen (AUC:88.5%, Accuracy:93% in GC). To find common molecular mechanisms of the miRNAs, their common gene targets were predicted using online databases such as miRWalk, miRDB, and Targetscan. Functional and enrichment analyzes were performed using Gene Ontology (GO) and Kyoto Database of Genes and Genomes (KEGG), as well as identification of protein–protein interactions (PPI) using the STRING database. Pathway analysis of the target genes revealed the involvement of several cancer-related pathways including miRNA mediated inhibition of translation, regulation of gene expression by genetic imprinting, and the Wnt signaling pathway. Survival and ROC curve analysis showed that the expression levels of hsa-miR-21, hsa-miR-133a, hsa-miR-146b, and hsa-miR-29c were associated with higher mortality and potentially earlier detection of GC patients. A panel of dysregulated miRNAs that may serve as reliable biomarkers for gastric cancer were identified using machine learning, which represents a powerful tool in biomarker identification.

## Introduction

Gastric cancer (GC) has a low 5-year survival rate in part due to misdiagnosis in the early stages of the disease and its recurrence, which makes this cancer one of the most prevalent cancers and the leading cause of cancer-related mortality^1^. Misdiagnosis of the GC in the early stages, when no symptoms are apparent, and only the accumulation of genetic changes occurs at the molecular level, brings a heavy financial and physical burden to patients thus emphasizing the need for a clear understanding of the underlying molecular pathways^2^. Among the components of this molecular network are the miRNAs, "short-length post-transcriptional regulators," which are part of the dynamic genome changes that act in the initiation and progression of cancers such as GC^3,4^. Recent advances in genome-wide sequencing and the use of bioinformatics tools to analyze their data have led to new roads to further identifying components and their functions in this intertwined molecular network. Due to the complex nature of the disease, the current biomarkers lack the necessary power for accurate diagnosis and prognosis, as a result, more studies have been directed towards the discovery of computational methods such as machine learning to predict the miRNA-disease relationship and their use in cancer diagnosis^5^. In a recent study, the use of Boruta's machine learning algorithm in a GEO dataset of gastric cancer patients introduced 30 miRNAs with high biomarker power, and finally, hsa-miR-1343-3p has been introduced as a valuable diagnostic biomarker^6^. In another study, Rehman et al. investigated clinically validated miRNAs, using machine learning. The results not only confirmed their concept but also indicated the potential of miRNA as diagnostic biomarkers in breast cancer and introducing machine learning as a powerful tool in functional studies^7^. Utilizing data from the study of human tumors in large cohorts, the TCGA project has developed an extensive catalog of genetic changes associated with these tumors, make cancer genome and transcriptome uncovered and shed new light on the diagnosis, treatment, and prognosis of cancer, as in gastric cancer with the help of accurate classification of gastric adenoma cancers into four subgroups, (EBV-positive tumors (EBV), microsatellite unstable tumors (MSI), genomically stable tumors and chromosomally unstable tumors (CIN) has led to improved clinical diagnosis and treatment in this cancer^8^. This study aimed to find differentially expressed miRNAs (demiRs) in gastric cancer which can act as an important biomarker for early detection.

## Material and methods

### Retrieval of genome data

Raw data from clinical information and sequencing of 576 stomach cancer were downloaded from the TCGA database (https://tcga-data.nci.nih.gov/), an available dataset that catalogs cancer-causing genomic changes^8^.

### Data preprocessing and identification of DemiRs

Processing and analyzing of raw data were accomplished using R software (version 4.2.0, Limma package, https://www.r-project.org/ ), to identify differentially expressed miRNAs (demiRs), adj. p < 0.05 and log|FC|> 1.5 were set as the threshold.

### Identifying prognostic biomarkers

The association between the expression level of previously identified demiRs and the overall survival rate and outcome of the patients was assessed by log-rank analysis (p < 0.05). To define and visualize miRNAs with prognostic ability, the Kaplan Meier was used. Survival, survminer, and ggplot2 R packages were used for this purpose.

### Identifying predictive biomarkers

Machine learning is defined as the intelligent analysis and design of models with the ability to find features with significant impact and discover the relationships between them based on algorithms and mass data analysis, which has comparable capabilities with human experts^9^. One of its several applications is in medical sciences to identify biomarkers with significant diagnostic value for various diseases as in the wide range of cancers such as colorectal cancer, breast cancer, gastric cancer, as well as cardiovascular disease, Alzheimer’s disease, and etc.^5,10–13^. In this study, we used machine learning to find miRNAs with diagnostic and predictive values. For this purpose, in the processing step, important features were identified using heatmap analysis, and five methods (SVM, DTS, RF, Logistic Regression, KNN) were used for classification; in the next step, all of these methods were compared with four different metrics (accuracy, f1score, ROC_curve, and confusion matrix) to find the most accurate algorithm. The algorithms were used in this study to measure the diagnostic ability of demiRs in GC which will be briefly mentioned in the following. One of the methods of evaluating the performance of binary classification is the "Receiver Operating Characteristic" or ROC curve. The efficiency of "Binary Classifier" algorithms is usually measured by indicators called "Sensitivity" or "Recall" but in the ROC chart, both of these indicators are combined and displayed as a curve. In the current study, the ROC curve is used to evaluate the efficiency diagnostic of selected hub miRNAs using R 4.2.2's combioROC package. For this purpose, sensitivity, specificity, cut-off value, positive predictive value, negative predictive value, and area under the ROC curve were measured. A support vector machine (SVM) algorithm is used for the classification of data points as well as regression in machine learning. SVMs have played an important role in a wide variety of biological applications, for example, the classification of microarray gene expression profiles in tumor and normal samples to find suitable diagnostic or prognostic biomarkers^14^. The K-Nearest Neighbors (KNN) algorithm is one of the simplest and at the same time the best algorithms used in supervised learning in the field of machine learning which considers the distance in classification and uses both “Regression” and “Classification” issues^15^. One of the methods of classification in “Supervised Machine Learning” is logistics regression. This method of regression, relies on an odd ratio for calculation. This type of analysis can help predict the probability of occurring an event or choice^16^. DTs: A decision tree is a tool for making a more appropriate decision in a way that gives a tree structure or hierarchical structure to decisions and their results. The structure of this tree can also be based on chance and probability, so choosing any decision randomly can bring risks or benefits^17^. Random Forest is an easy-to-use machine learning algorithm that often provides excellent results even without adjusting its meta-parameters. This algorithm is considered one of the most used machine learning algorithms due to its simplicity and usability, both for "Classification" and "Regression"^14^.

### Machine learning performances

The accuracy parameter is the most commonly used, basic, and simple measure of the quality of a category, and it is the amount of correct recognition of the category in a total of two categories^18^. "System performance characteristic curve" (Receiver Operating Characteristic | ROC) is an appropriate evaluation index to ensure the validity of the results based on sensitivity and specificity. (Area Under Curve) is used as a measure to evaluate the performance of the category^19^. When the accuracy of the diagnosis of a category is more important compared to the accuracy of the overall diagnosis, the concept of "Confusion Matrix" comes to our aid. confusion matrix can give you a better idea of what your classification model is getting right and what types of errors it is making^20^.

### Validation of candidate microRNAs in datasets

In order to validated the candidate miRNAs which resulted by machine learning algorithms, we validated them using online web server such as, Global Data Assembly Centres (GDAC) (/https://gdac.broadinstitute.org/) and dbDMEC (https://www.biosino.org/dbDEMC/index ) webtools which contains the Differentially Expressed MiRNAs in human Cancers based on public repositories including Gene Expression Omnibus (GEO), Sequence Read Archive (SRA), ArrayExpress and The Cancer Genome Atlas (TCGA).

### miRNA-target prediction

Using online databases for simple prediction of miRNA-targets such as miRwalk (http://mirwalk.umm.uni-heidelberg.de/)^21^, miRDB (http://www.mirdb.org/)^22^, and Target scan (https://www.targetscan.org/vert_80/)^23^, target genes of significant validated up and down expressed miRNAs were predicted.

### PPI network analysis

Search Tool for the Retrieval of Interacting Genes (STRING; https://string-db.org/)^24^ with confidence scores ≥ 0.9 as a threshold was used to find an interaction between the identified miRNAs’ targets.

### Functional and pathway enrichment analysis

Using functional annotation tools such as (GO; http://geneontology.org/)^25^which annotate genes considering three aspects biological functions (BF), molecular functions (MF), and cellular components (CC), and Kyoto Encyclopedia of Genes and Genomes (KEGG; https://www.genome.jp/kegg/)^26^, we clarified the functional and enrichment analysis of the candidate genes.

### Correlation analysis

Correlation matrix analyses were then performed between miRNAs and clinicopathological characteristics including, gender, age, stage, and prior malignancy using Spearman’s correlation method.

### Construction of miRNA–mRNA network

**Figure 1:**
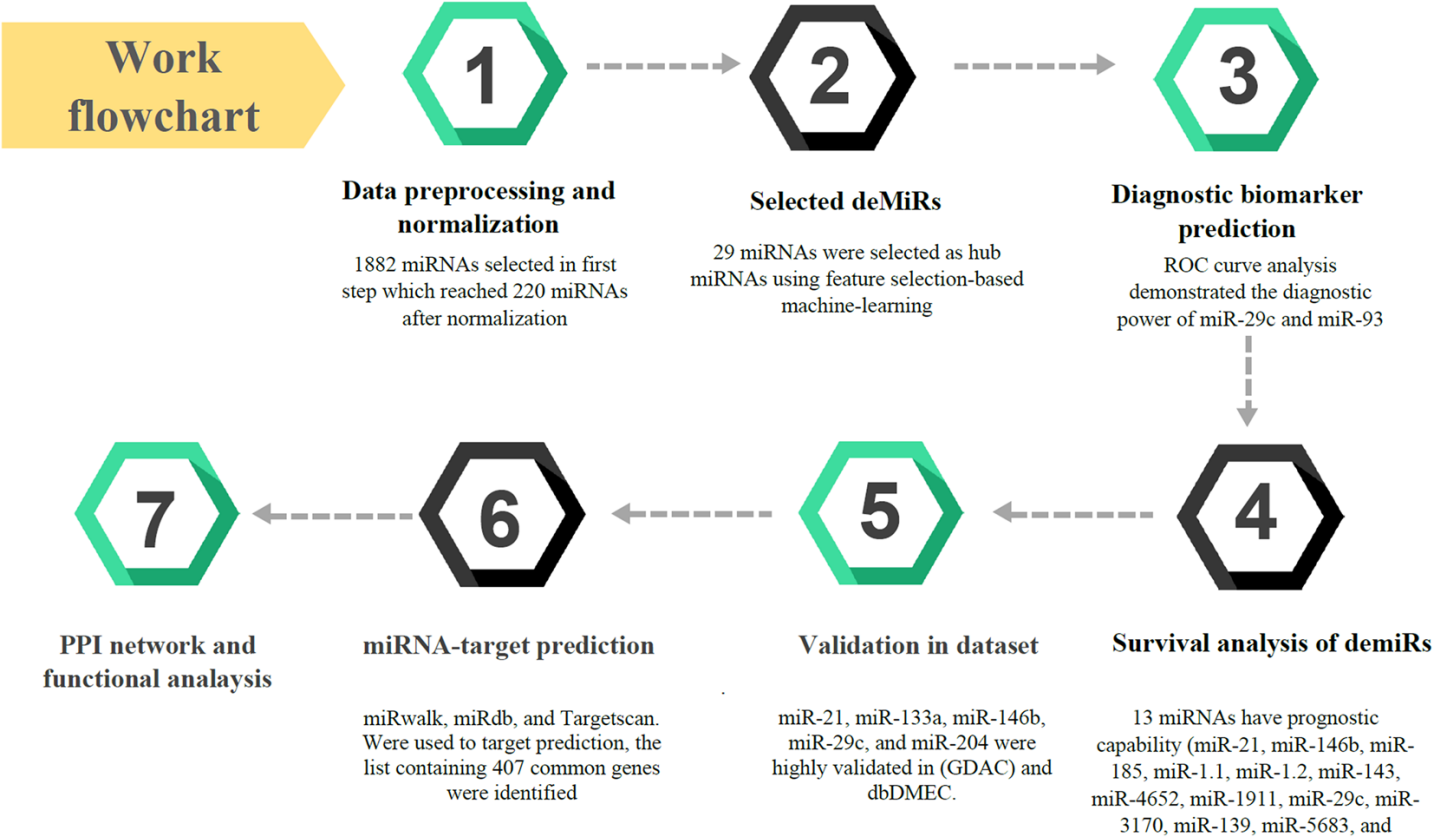
Flowchart of methodology performed in this research.

To obtain a clear visualization of the miRNAs-mRNAs interaction network, Cytoscape software (version 3.9.1) (https://cytoscape.org/)^27^ was used. Highly interconnected miRNAs and genes were then demonstrated. The flowchart of the methodology used in the current study is demonstrated in Fig. 1.

## Results

### Demographic information

The clinicopathological information of 348 (64.9%) men and 188 (35.1%) women included in this study were downloaded from TCGA and summarized in Table 1. The average age was 65.3 years and around 250 (46.6%) had advanced gastric cancer.

**Table 1:** Demographic information.

Demographic variables	No. of patients (%)/Mean ± SD
Patients	464
Mean age (years, mean ± SD)	65.33 ± 12.091
Gender	
Female	188 (35.1)
Male	348 (64.9)
Stage	
1	78 (14.6)
2	180 (33.6)
3	198 (36.9)
4	52 (9.7)
Prior malignancy	
Yes	13 (2.4)
No	523 (97.6)

### Correlation analysis

**Figure 2:**
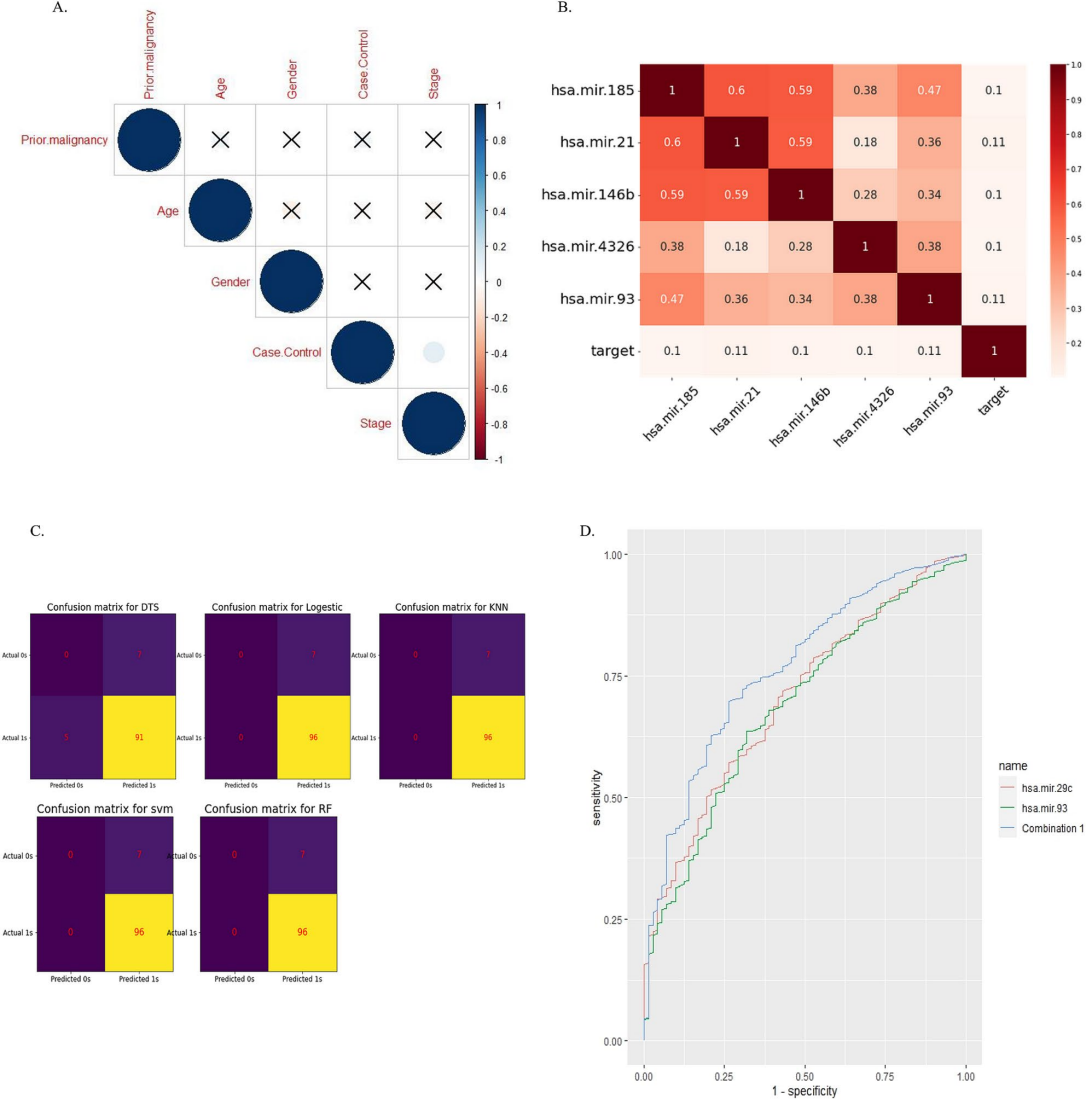
(**A**) correlation analysis using ggcorpot package, R software v 4.2; (**B**) Heat map analysis used to demonstrate important features. (**C**) The confusion matrix was used to compare the different machine-learning algorithms, figure B&C was plotted using Python v3.7. (**D**) ROC curve analysis revealed biomarker potency of miR-29c alone and in combination with miR-93 using R 4.2.2's combioROC package.

Of the clinicopathological data, only the stage of disease was significantly associated with cancer. The significant criterion for measuring association was considered a *p*-value of < 0.05 (Fig. 2A).

### Data collection

As mentioned in the material & method section, the source of the Raw data from clinical information and sequencing was the TCGA database. Based on the mentioned criteria, 536 samples were selected for further studies of which about 465 were related to patients with GC and 72 were related to age and sex-matched control.

### Data preprocessing and identification of differentially expressed miRNAs (DeMiRs)

**Figure 3:**
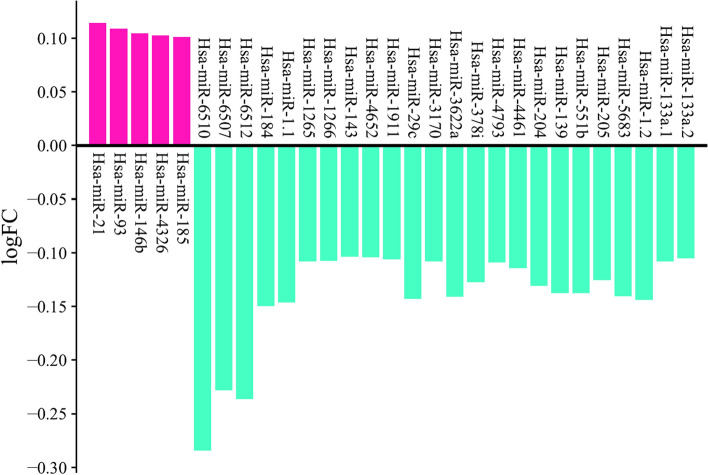
29miRNAs obtained from feature selection-based machine learning based on logFC.

The dataset included 1882 miRNAs, which were reduced to 220 miRNAs after normalization using the Limma package, R software. In the processing step using a heat map, the most important features were selected (Fig. 2B) and classified using machine learning algorithms. five algorithms (SVM, dts, rf, logistic regression, and knn) were then examined with four different metrics (accuracy, f1score, ROC_curve, and confusion matrix), and finally, according to the score obtained from these four metrics, the SVM algorithm was selected as the most accurate algorithm. (**DTS,** Accuracy: 88%, AUC = 47%; **RANDOM FOREST,** Accuracy: 93%, AUC = 39.5%; **SVM,** Accuracy: 93%, AUC = 88.5%; **KNN,** Accuracy: 93%, AUC = 41.7%; **LOGISTIC,** Accuracy: 93%, AUC = 88%). The confusion matrix can also be seen in Fig. 2C. As a result, a list of 29 miRNAs with five significant up and 24 significant down expressions in gastric cancer opted for further analysis (Table 2) Fig. 3.

**Table 2:** List of sign up and down-expressed miRNAs in gastric cancer.

	Fold change
Upregulated miRNAs	
Hsa-miR-21	0.114061
Hsa-miR-93	0.108749
Hsa-miR-146b	0.104449
Hsa-miR-4326	0.1022
Hsa-miR-185	0.100701
Downregulated miRNAs	
Hsa-miR-6510	− 0.284227
Hsa-miR-6507	− 0.227782
Hsa-miR-6512	− 0.236185
Hsa-miR-184	− 0.149492
Hsa-miR-1.1	− 0.146
Hsa-miR-1265	− 0.107646
Hsa-miR-1266	− 0.107325
Hsa-miR-143	− 0.103477
Hsa-miR-4652	− 0.104071
Hsa-miR-1911	− 0.106134
Hsa-miR-29c	− 0.142613
Hsa-miR-3170	− 0.107759
Hsa-miR-3622a	− 0.140976
Hsa-miR-378i	− 0.127142
Hsa-miR-4793	− 0.109031
Hsa-miR-4461	− 0.114218
Hsa-miR-204	− 0.130772
Hsa-miR-139	− 0.137331
Hsa-miR-551b	− 0.137459
Hsa-miR-205	− 0.125177
Hsa-miR-5683	− 0.140301
Hsa-miR-1.2	− 0.143739
Hsa-miR-133a.1	− 0.107877
Hsa-miR-133a.2	− 0.104760

### ROC curve analysis for identification of diagnostic biomarkers

The findings of the ROC curve analysis demonstrated the diagnostic power of hsa-miR-29c (AUC of 0.7, with a sensitivity of 0.5 and specificity of 0.8, and cutoff of 0.88) which is improved when combined with hsa-miR-93 (combination had an AUC of 0.76, the sensitivity of 0.69 and specificity of 0.73 and cutoff of 0.86) (Fig. 2D).

### Survival analysis of demiRs

**Figure 4:**
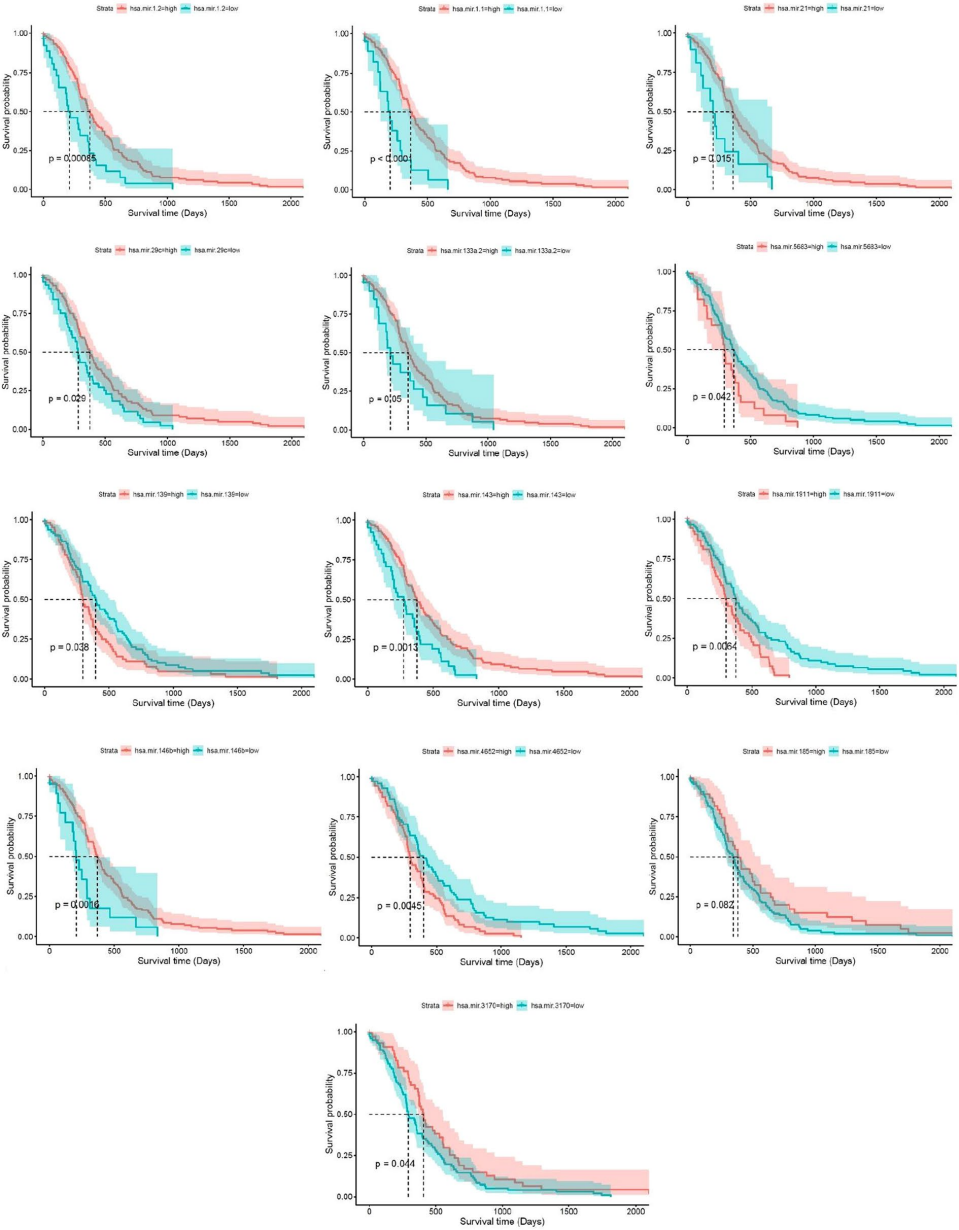
Kaplan Meier visualization of Identified prognostic biomarkers using Survival, survminer, and ggplot2 R packages from R software v4.2.2.

Survival analysis of demiRs was performed using SPSS version 20, and the *p*-value was considered < 0.05. the results demonstrated that 13 miRNAs (Hsa-miR-21, Hsa-miR-146b, Hsa-miR-185, Hsa-miR-1.1, Hsa-miR-1.2, Hsa-miR-143, Hsa-miR-4652, Hsa-miR-1911, Hsa-miR-29c, Hsa-miR-3170, Hsa-miR-139, Hsa-miR-5683, and Hsa-miR-133a.2) have the prognostic capability (Fig. 4).

### Validation of candidate microRNAs in datasets

**Figure 5:**
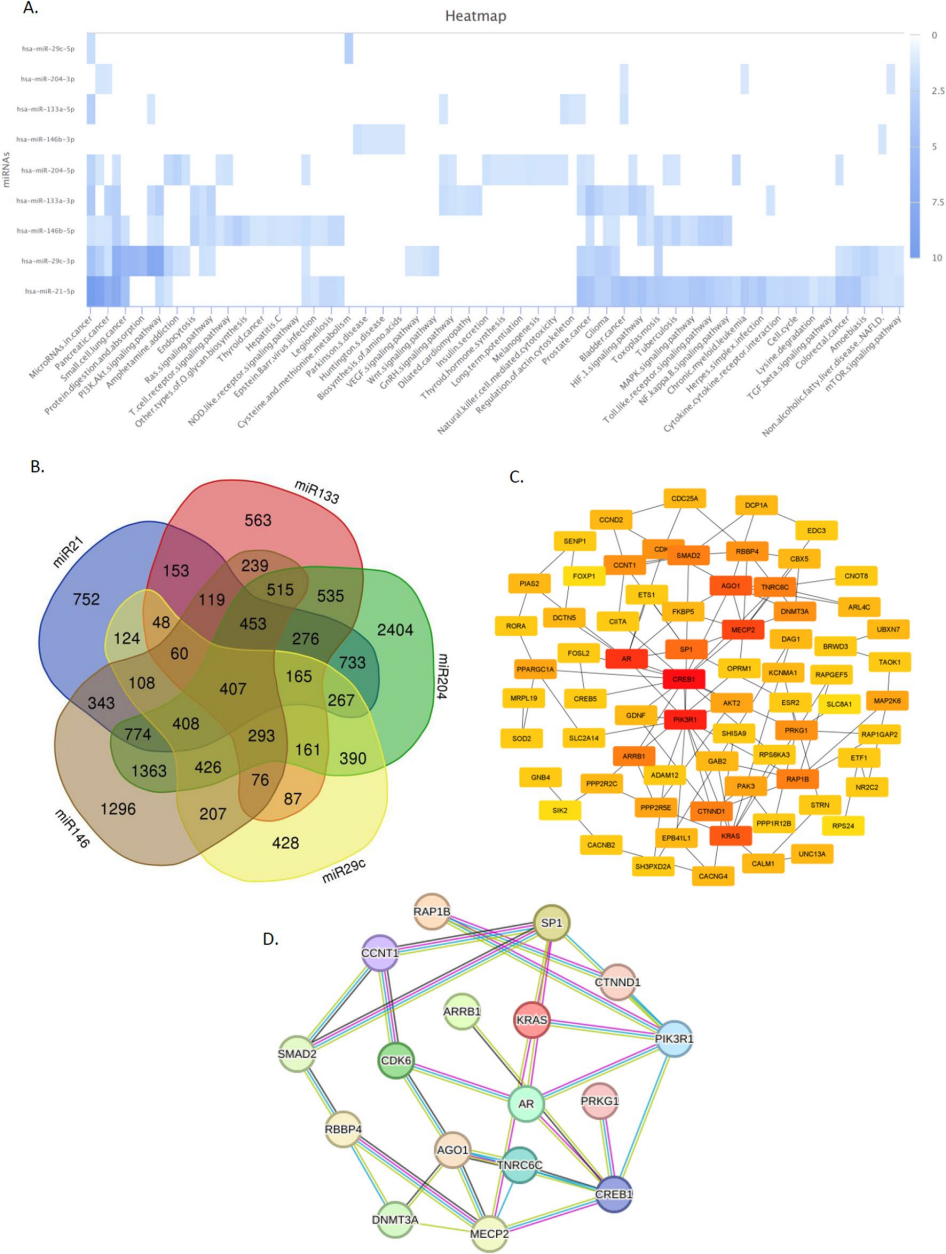
(**A**) Heat map of highly validated miRNAs and pathway they involved using miRPathDB v2.0 (https://mpd.bioinf.uni-sb.de/heatmap_calculator.html?organism=hsa) (**B**) candidate miRNAs and their common target genes using Venn diagram online database (https://bioinformatics.psb.ugent.be/webtools/Venn/); (**C**) 100 highly scored genes based on degree were selected using Cytohubba tools (https://cytoscape.org/ cytoscape version3.9.1) (**D**) miRNA-target genes; Hub genes reanalyzed by string database consist of 100 nodes and 223 edges. We set the highest confidence score of 0.9 and hide disconnected nodes in the network (https://string-db.org/).

Among 29 candidate microRNAs resulted from machine learning algorithms, using online web servers mentioned in material and method section, the expression levels of hsa-miR-21, hsa-miR-133a, hsa-miR-146b, hsa-miR-29c, and hsa-miR-204 were highly validated in (EXP00118(GSE28700), EXP00131 (GSE23739), EXP00230 (GSE26595), EXP00268, EXP00270 (GSE54397) EXP00326 (GSE31568), EXP00337 (GSE59856), EXP00404, EXP00460 (GSE93415), EXP00524 (GSE106817), EXP00405, EXP00118(GSE28700), EXP00406, EXP00666, EXP00444 (GSE78775), EXP00476 (GSE99415), EXP00316 (GSE77380), and EXP00175(GSE33743)) supplementary file 1, heatmap analysis performed using miRPathDB online server (https://mpd.bioinf.uni-sb.de/ , Fig. 5A).

### miRNA-target prediction

The miRNA-target prediction was accomplished using several databases, that included: miRwalk, miRdb, and Targetscan. Using a Venn diagram online database, the list containing 407 common genes were identified (Fig. 5B).

### Protein–protein interaction network analysis

Candidate genes predicted in the previous step were submitted to the STRING database to build a PPI network based on criteria mentioned in the material and method. To obtain hub genes with essential roles, the PPI network was then imported and visualized by Cytoscape software. 100 highly scored genes based on degree were selected using Cytohubba tools (Fig. 5C). Finally, the hub genes were imported into the string database to reanalyze the PPI network (Fig. 5D).

### Functional analysis

**Figure 6:**
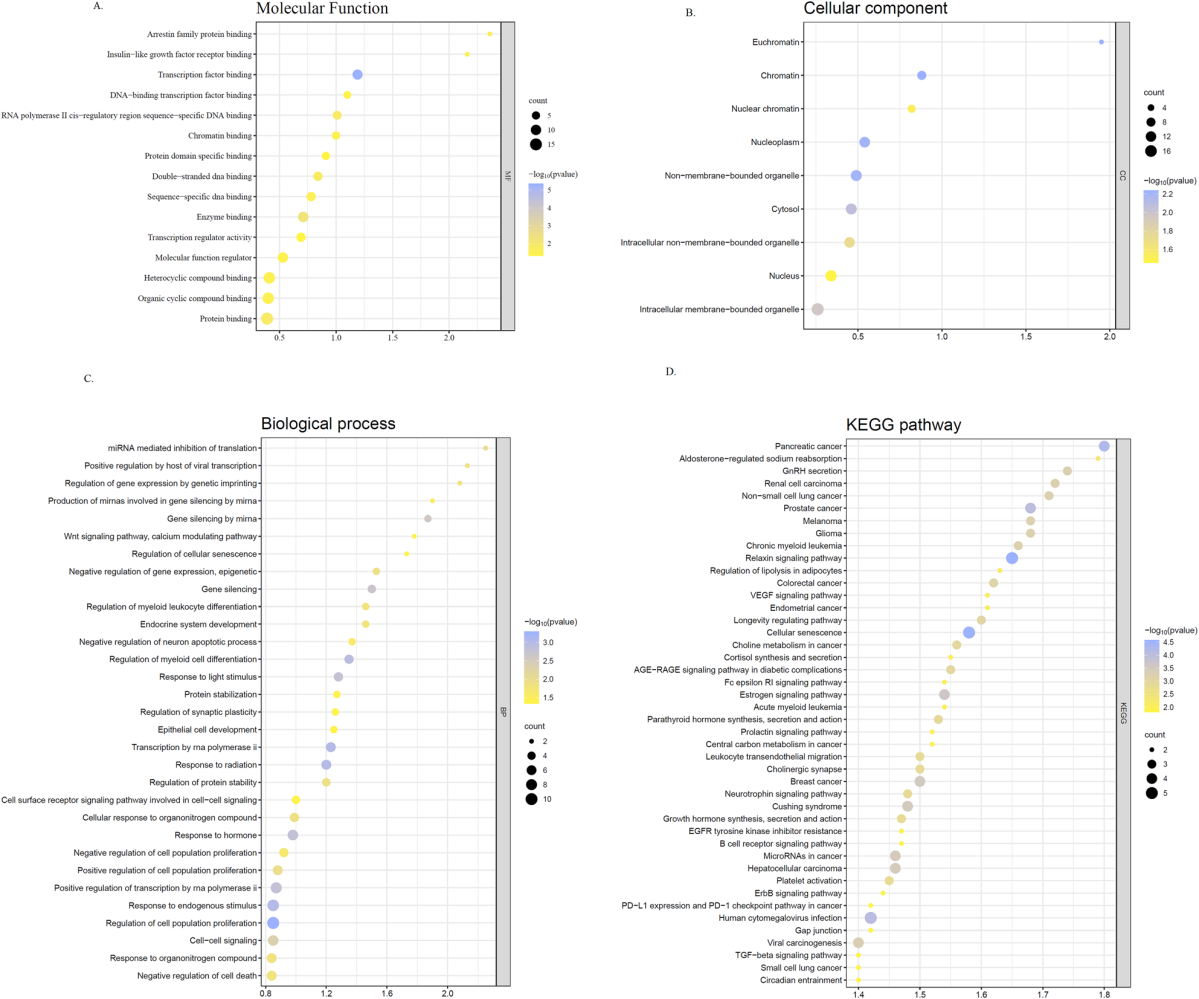
Functional enrichment analysis of target genes based on gene ontology regarding (**A**) molecular function, (**B**) cellular component (**C**) biological process. (**D**) KEGG pathway analysis. All the analysis was plotted using SRplot http://www.bioinformatics.com.cn/srplot, an online platform for data analysis and visualization.

To reveal the role of selected hub genes, enrichment analysis was undertaken using R software. The results demonstrated that hub genes transcription factor binding, enzyme binding, RNA polymerase II cis-regulatory region sequence-specific DNA binding, protein binding, double-stranded DNA binding, arrestin family protein binding, sequence-specific DNA binding, and chromatin binding, regarding molecular function, The majority of genes were enriched in miRNA mediated inhibition of translation, Positive regulation by host of viral transcription, Regulation of gene expression by genetic imprinting, Production of miRNAs involved in gene silencing by miRNA Gene silencing by miRNA, Wnt signaling pathway, calcium modulating pathway, Regulation of cellular senescence, Negative regulation of gene expression, and epigenetic Gene silencing concerning the biological process. Chromatin, Euchromatin, Nucleoplasm, Non-membrane-bounded organelle and Cytosol were top enriched cellular components. Identification of significant signaling pathways using the KEGG database shows that candidate hub genes were mainly involved in Glioma, Melanoma, Prostate cancer, Non − small cell lung cancer, Renal cell carcinoma, GnRH secretion, Aldosterone − regulated sodium reabsorption, and Pancreatic cancer (Fig. 6).

## Discussion

Gastric cancer is a heterogeneous disease with a poor prognosis^28^. Despite many advances in treatment including the use of adjuvant therapies such as surgery, chemotherapy, radiotherapy, and targeted therapy, the overall 5-year survival rate of the disease is still around 20–30% due to the diagnosis of patients in advanced stages of the disease^29^. Therefore, it is important to identify biomarkers that can detect the disease in its early stages and also identify its likely prognosis. MicroRNAs (miRNAs) are a small member of the non-coding RNAs family that control various post-transcriptional processes and possess important role in carcinogenesis including angiogenesis, drug resistance, and cancer metastasis^30,31^. In addition, the importance of miRNA-mRNA networks in activating or inhibiting many cancer-related molecular signaling pathways has recently been observed^31,32^. Recently, with the development of bioinformatics as well as the aid of machine learning algorithms, different forms of next-generation sequencing are increasingly being used to detect biomarkers with a role in early detection, treatment, and prognosis of cancer^33–35^. Machine learning approaches have gained popularity due to their predictive power of diagnostic and prognostic biomarkers. Studies also suggest the role of machine learning in feature selection such as miRNAs as an initial option when there is no prior knowledge^36,37^. In the present study, TCGA data analysis of stomach cancer using machine learning algorithms identified 29 miRNA candidates. Even though machine learning offers different algorithms for selecting a gene as a biomarker, none of them alone and without clinical validation can guarantee the true power of a biomarker and the reproducibility of the results requires more investigations and validations^38^. In order to overcome the limitations of machine learning, we validated the results obtained from it in different datasets such as GEO, TCGA, and GDAC, and among the 29 miRNAs found, hsa-miR-21, hsa-miR-133a, hsa-miR-146b, hsa-miR-29c, and hsa-miR-204 were among the most valid miRNAs in the investigated datasets.

Subsequent studies were designed to determine the role of these demiRs in gastric cancer through joint bioinformatics and prognosis analyses. In the first step, we explored the genes targeted by the five validated demiRs using online web server such as miRwalk, miRdb, and Targetscan. PPI network and cytohubba were then identified top 100 high-scored genes of each group of genes and functional and enrichment analyses were performed. GO and KEGG analysis determined that DEGs were mainly involved in cancer-related pathways. in concert, it was demonstrated that these feature miRNAs are highly correlated with miRNA mediated inhibition of translation , Regulation of gene expression by genetic imprinting, Production of miRNAs involved in gene silencing by miRNA Gene silencing by miRNA, Wnt signaling pathway, calcium modulating pathway, Regulation of cellular senescence, Negative regulation of gene expression based on functional enrichment analysis.

Survival analysis also reveal the prognostic capability of hsa-miR-21, hsa-miR-146b, hsa-miR-185, hsa-miR-1.1, hsa-miR-1.2, hsa-miR-143, hsa-miR-4652, hsa-miR-1911, hsa-miR-29c, hsa-miR-3170, hsa-miR-139, hsa-miR-5683, and hsa-miR-133a.2. On the other hand, ROC curve analysis demonstrated the diagnostic power of hsa-miR-29c in combination with hsa-miR-93. From these data, we can consider hsa-miR-21, hsa-miR-133a, hsa-miR-146b, and hsa-miR-29c a reliable biomarker panel. In a study by Larki et.al, the expression level of miR-21 and miR-93 were investigated in GC patients with the aim of finding a panel of diagnostic signature, it was demonstrated that the over-expression of these miRNAs were highly correlated with early diagnosis and prognosis of this cancer^39^. In another study conducted by CHAN et.al the diagnostic potency of miR-21 were detected, although they claimed that this miRNA can’t serve as prognostic biomarker^40^. Simonian considered miR-21 as both diagnostic and prognostic biomarker and suggested that it could be higher diagnostic potential compare to CA-19-9^41^. Also several study mentioned miR-133a as a tumor suppressor miRNA which inhibit the GC growth and exert its effect via different target genes such as Sp1, IGF1R, TCF4 and etc^42–44^. Although the effect of has-miR-146b is less investigated in GC, in the study, the over-expression of this miRNA is demonstrated in GC patients, which exert its effect on NOVA1 and associated with poor prognosis^45^. It is worth noting that based on the results of this study hsa-miR-29c have a dual biomarker potency and serves as both prognostic and diagnostic biomarker in GC. Although several studies mentioned the role of this miRNA as a diagnostic biomarker in GC, as far as our knowledge is concerned there isn’t any study concerning its prognostic capability. On the other hand, the machine learning method used in this study was demonstrated three significant down-regulated miRNAs has-miR-6510, has-miR-6507, has-miR-6512 in gastric cancer which recently gained attention in gastric cancer. Li et.al showed that miR-6512 downregulated in gastric cancer patients and seems to have a correlation with skin manifestations and fibrosis^46^. Moreover, a study by Ding demonstrated that miR-6507 was among top five down expressed miRNAs in GC and may serve as a good predictive biomarker^47^. Another study introduced miR-6510 as a potential prognostic biomarker in GC patients^48^. Although their role is not fully identified and need further investigation, these miRNAs could play an important role in GC.

## Conclusion

In conclusion, 29 miRNAs were identified using machine learning algorithms, subsequent analyzes showed a panel of four miRNAs including hsa-miR-21, hsa-miR-133a, hsa-miR-146b, and hsa-miR-29c with high diagnostic and prognostic power, which was validated by several datasets. This study emphasizes the importance of machine learning as an alternative option for predicting biomarkers in gastric cancer. However, for the purpose of further validation, it is suggested that candidate miRNAs will be analyzed in several cohorts in a laboratory manner.

## Supplementary Information

Supplementary Information 1.

Supplementary Information 2.

Supplementary Information 3.

## Data Availability

The datasets used and/or analyzed during the present study are available from the corresponding author upon reasonable request.
