# Supplementary material for: Machine learning algorithms reveal potential miRNAs biomarkers in gastric cancer
Source: Sci Rep. 2023 Apr 15;13:6147. doi: 10.1038/s41598-023-32332-x (PMC10105697; doi:10.1038/s41598-023-32332-x)
Supplement: Supplementary file 3 — Supplementary Information 3. [file 41598_2023_32332_MOESM3_ESM.pdf]

**######Machine learning Codes used to select miRNAs**

```
#import libraries...

import pandas as pd

import numpy as np

import matplotlib.pyplot as plt

import seaborn as sns

from sklearn.model_selection import train_test_split

from sklearn.naive_bayes import GaussianNB

from sklearn import metrics

from sklearn.model_selection import train_test_split

from sklearn.model_selection import KFold

from sklearn.model_selection import cross_val_score

from sklearn import preprocessing

from sklearn.preprocessing import PolynomialFeatures

from sklearn.metrics import roc_curve

from sklearn.metrics import roc_auc_score

from matplotlib.axis import Axis

import matplotlib.ticker as ticker

from sklearn.neighbors import KNeighborsClassifier

from sklearn.linear_model import LogisticRegression

from sklearn.naive_bayes import BernoulliNB

from sklearn.naive_bayes import MultinomialNB

from sklearn.tree import DecisionTreeClassifier

from sklearn.svm import SVC

from sklearn.ensemble import RandomForestClassifier

from sklearn.metrics import classification_report

from sklearn.metrics import confusion_matrix

from sklearn.metrics import precision_recall_curve

from sklearn.metrics import f1_score
```

```
from numpy import arange
from numpy import argmax
from sklearn.metrics import precision_score
from sklearn.metrics import recall_score
from sklearn.model_selection import GridSearchCV, StratifiedKFold
from collections import Counter
from numpy import where
from imblearn.under_sampling import OneSidedSelection
from imblearn.over_sampling import RandomOverSampler
from imblearn.under_sampling import RandomUnderSampler
from sklearn.preprocessing import StandardScaler
from sklearn.decomposition import PCA
from lifelines import KaplanMeierFitter
from sklearn.ensemble import VotingClassifier
from sklearn.ensemble import AdaBoostClassifier
from sklearn.ensemble import GradientBoostingClassifier
import xgboost as xgb

data=pd.read_csv('D:/python/data set/final mir stad 2.csv')
data
df_cancer_sto=pd.DataFrame(data)
df_cancer_sto
df_cancer_sto.drop(['barcode','sample','shortLetterCode','ajcc_pathologic_stage','days_to_death','primary_diagnosis'],axis=1,inplace=True)
df_cancer_sto
df_cancer_sto['prior_malignancy'].replace('no',0,inplace=True)
df_cancer_sto['prior_malignancy'].replace('yes',1,inplace=True)
df_cancer_sto['vital_status'].replace('Dead',1,inplace=True)
df_cancer_sto['vital_status'].replace('Alive',0,inplace=True)
```

```
df_cancer_sto['treatments_pharmaceutical_treatment_or_therapy'].replace('no',0,inplace=True)
df_cancer_sto['treatments_pharmaceutical_treatment_or_therapy'].replace('yes',1,inplace=True)
df_cancer_sto['treatments_radiation_treatment_or_therapy'].replace('no',0,inplace=True)
df_cancer_sto['treatments_radiation_treatment_or_therapy'].replace('yes',1,inplace=True)
df_cancer_sto['treatments_pharmaceutical_treatment_or_therapy'].replace('no',0,inplace=True)
df_cancer_sto['treatments_pharmaceutical_treatment_or_therapy'].replace('yes',1,inplace=True)
df_cancer_sto['synchronous_malignancy'].replace('No',0,inplace=True)
df_cancer_sto['synchronous_malignancy'].replace('Yes',1,inplace=True)
df_cancer_sto['target'].replace('Solid Tissue Normal',0,inplace=True)
df_cancer_sto['target'].replace('Primary solid Tumor',1,inplace=True)
df_cancer_sto['gender'].replace('female',0,inplace=True)
df_cancer_sto['gender'].replace('male',1,inplace=True)
df_cancer_sto.describe()
df_cancer_sto.dropna(inplace=True)
df_cancer_sto
df_cancer_sto.drop_duplicates(subset='patient',inplace=True)
df_cancer_sto
df_cancer_sto.drop('patient',axis=1,inplace=True)
plt.figure(figsize=(28,8))
ax = sns.countplot(x="target", data=df_cancer_sto)
plt.xticks(fontsize=20)
plt.yticks(fontsize=20)
plt.xlabel("gene",fontsize=20)
plt.ylabel("Count",fontsize=20)
plt.title("name of Genes",fontsize=30)
plt.grid()
c=df_cancer_sto[df_cancer_sto.columns[:]].corr()['target'][:]
df2=pd.DataFrame(c)
df2[df2['target']>0.1]
```

```
df2=pd.DataFrame(c)

df2[df2['target']<-0.1]

#making a data frame with selected features.

df_important=pd.DataFrame(df_cancer_sto,columns=['hsa.mir.185', 'hsa.mir.21', 'hsa.mir.146b',
'hsa.mir.4326',
      'hsa.mir.93', 'target'])

df_important

#corrolation table

plt.figure(figsize=(12,8))

cor = df_important.corr()

sns.heatmap(cor, annot=True, cmap=plt.cm.Reds,annot_kws={"size": 15})

plt.yticks(rotation=0,fontsize=20)

plt.xticks(fontsize=17,rotation=45)

plt.show()

df_kaplan=pd.DataFrame(data,columns=['days_to_death','hsa.mir.185', 'hsa.mir.21', 'hsa.mir.146b',
'hsa.mir.4326',
      'hsa.mir.93', 'target'])

df_kaplan.dropna(inplace=True)

df_kaplan

kmf = KaplanMeierFitter()

df_kaplan2=pd.DataFrame(data,columns=['days_to_death','vital_status'])

df_kaplan2.dropna(inplace=True)

df_kaplan2.reset_index(drop=True)

df_kaplan2['vital_status'].replace('Dead',1,inplace=True)

kmf.fit(durations = df_kaplan2['days_to_death'], event_observed = df_kaplan2['vital_status'],label =
"DEATH")

kmf.plot_survival_function()


#LETS MAKE A MODEL!

df_important2=df_important.drop(['target'],axis=1)
```

```
df_important2
x=pd.DataFrame(df_important2).values
y=df_important.target.values.reshape(-1,1)
x_train,x_test,y_train,y_test=train_test_split(x,y,test_size=0.25,random_state=0)
model_dts = DecisionTreeClassifier(random_state=0)
model_rf = RandomForestClassifier(random_state=0)
model_svm=SVC(random_state=0)
model_svm_roc=SVC(random_state=0,probability=True)# for drawing roc plot
model_logistic = LogisticRegression(solver='liblinear',penalty='l2',max_iter=1000)
model_knn = KNeighborsClassifier()
model_dts.fit(x_train, y_train.ravel())
model_rf.fit(x_train, y_train.ravel())
model_svm_roc.fit(x_train, y_train.ravel())
model_svm.fit(x_train, y_train.ravel())
model_logistic.fit(x_train, y_train.ravel())
model_knn.fit(x_train, y_train.ravel())
#for modeling
y_pred_dts = model_dts.predict(x_test)
y_pred_dts_proba=model_dts.predict_proba(x_test)[:,1]# for drawing roc plot
y_pred_rf = model_rf.predict(x_test)
y_pred_rf_proba=model_rf.predict_proba(x_test)[:,1]# for drawing roc plot
y_pred_svm = model_svm.predict(x_test)
y_pred_svm_roc = model_svm_roc.predict_proba(x_test)[:,1]# for drawing roc plot
y_pred_logistic = model_logistic.predict(x_test)
y_pred_logistic_proba=model_logistic.predict_proba(x_test)[:,1]# for drawing roc plot
y_pred_knn = model_knn.predict(x_test)
y_pred_knn_proba=model_knn.predict_proba(x_test)[:,1]# for drawing roc plot
#accuracy and f1score
print('Accuracy for DTS:',metrics.accuracy_score(y_test,y_pred_dts))
```

```
print('F1_SCORE for DTS:',metrics.f1_score(y_test,y_pred_dts,average='micro'))
print('#')
print('Accuracy for RF:',metrics.accuracy_score(y_test,y_pred_rf))
print('F1_SCORE for RF:',metrics.f1_score(y_test,y_pred_rf,average='micro'))
print('#')
print('Accuracy for SVM:',metrics.accuracy_score(y_test,y_pred_svm))
print('F1_SCORE for SVM:',metrics.f1_score(y_test,y_pred_svm,average='micro'))
print('#')
print('Accuracy for KNN:',metrics.accuracy_score(y_test,y_pred_knn))
print('F1_score for KNN:', metrics.f1_score(y_test,y_pred_knn,average='micro'))
print('#')
print('Accuracy for Logistic:',metrics.accuracy_score(y_test,y_pred_logistic))
print('F1_score for Logistic:', metrics.f1_score(y_test,y_pred_logistic,average='micro'))
#Confusion matrix
cm_dts = confusion_matrix(y_test, y_pred_dts)


fig,(ax0,ax1,ax2)=plt.subplots(1,3,figsize=(20,8))
ax0.imshow(cm_dts)
ax0.grid(False)
ax0.xaxis.set(ticks=(0, 1), ticklabels=('Predicted 0s', 'Predicted 1s'))
ax0.yaxis.set(ticks=(0, 1), ticklabels=('Actual 0s', 'Actual 1s'))
ax0.set_ylim(1.5, -0.5)
ax0.set_title('Confusion matrix for DTS',fontsize=20)
for i in range(2):
   for j in range(2):
      ax0.text(j, i, cm_dts[i, j], ha='center', va='center', color='red',fontsize=15)


cm_logestic = confusion_matrix(y_test, y_pred_logistic)
ax1.imshow(cm_logestic)
```

```
ax1.grid(False)
ax1.xaxis.set(ticks=(0, 1), ticklabels=('Predicted 0s', 'Predicted 1s'))
ax1.yaxis.set(ticks=(0, 1), ticklabels=('Actual 0s', 'Actual 1s'))
ax1.set_ylim(1.5, -0.5)
ax1.set_title('Confusion matrix for Logestic',fontsize=20)
for i in range(2):
   for j in range(2):
      ax1.text(j, i, cm_logestic[i, j], ha='center', va='center', color='red',fontsize=15)



cm_knn = confusion_matrix(y_test, y_pred_knn)
ax2.imshow(cm_knn)
ax2.grid(False)
ax2.xaxis.set(ticks=(0, 1), ticklabels=('Predicted 0s', 'Predicted 1s'))
ax2.yaxis.set(ticks=(0, 1), ticklabels=('Actual 0s', 'Actual 1s'))
ax2.set_ylim(1.5, -0.5)
ax2.set_title('Confusion matrix for KNN',fontsize=20)
for i in range(2):
   for j in range(2):
      ax2.text(j, i, cm_knn[i, j], ha='center', va='center', color='red',fontsize=15)

fig,(ax3,ax4)=plt.subplots(1,2,figsize=(10,8))
cm_svm = confusion_matrix(y_test, y_pred_svm)
ax3.imshow(cm_svm)
ax3.grid(False)
ax3.xaxis.set(ticks=(0, 1), ticklabels=('Predicted 0s', 'Predicted 1s'))
ax3.yaxis.set(ticks=(0, 1), ticklabels=('Actual 0s', 'Actual 1s'))
ax3.set_ylim(1.5, -0.5)
```

```
ax3.set_title('Confusion matrix for svm',fontsize=20)
for i in range(2):
    for j in range(2):
        ax3.text(j, i, cm_svm[i, j], ha='center', va='center', color='red',fontsize=15)



cm_rf = confusion_matrix(y_test, y_pred_rf)
ax4.imshow(cm_rf)
ax4.grid(False)
ax4.xaxis.set(ticks=(0, 1), ticklabels=('Predicted 0s', 'Predicted 1s'))
ax4.yaxis.set(ticks=(0, 1), ticklabels=('Actual 0s', 'Actual 1s'))
ax4.set_ylim(1.5, -0.5)
ax4.set_title('Confusion matrix for RF',fontsize=20)
for i in range(2):
    for j in range(2):
        ax4.text(j, i, cm_rf[i, j], ha='center', va='center', color='red',fontsize=15)




ax0.tick_params(axis='both', which='major', labelsize=12)
ax1.tick_params(axis='both', which='major', labelsize=12)
ax2.tick_params(axis='both', which='major', labelsize=12)
ax3.tick_params(axis='both', which='major', labelsize=12)
ax4.tick_params(axis='both', which='major', labelsize=12)
plt.show()
#auc curve
plt.figure(figsize=(12,8))
```

```
fpr_logistic, tpr_logistic, thresh_logistic = roc_curve(y_test, y_pred_logistic_proba, pos_label=1)

fpr_knn, tpr_knn, thresh_knn = roc_curve(y_test, y_pred_knn_proba, pos_label=1)

fpr_svm, tpr_svm, thresh_svm = roc_curve(y_test, y_pred_svm_roc, pos_label=1)

fpr_dts, tpr_dts, thresh_dts = roc_curve(y_test, y_pred_dts_proba, pos_label=1)

fpr_rf, tpr_rf, thresh_rf = roc_curve(y_test, y_pred_rf_proba, pos_label=1)



random_probs = [0 for i in range(len(y_test))]

p_fpr, p_tpr, _ = roc_curve(y_test, random_probs, pos_label=1)


auc_score_logistic = roc_auc_score(y_test, y_pred_logistic_proba)

auc_score_knn = roc_auc_score(y_test, y_pred_knn_proba)

auc_score_svm = roc_auc_score(y_test, y_pred_svm_roc,multi_class='ovr')

auc_score_dts = roc_auc_score(y_test, y_pred_dts_proba,multi_class='ovr')

auc_score_rf = roc_auc_score(y_test, y_pred_rf_proba,multi_class='ovr')



auc_logestic = metrics.roc_auc_score(y_test, y_pred_logistic_proba)

auc_knn = metrics.roc_auc_score(y_test, y_pred_knn_proba)

auc_svm = metrics.roc_auc_score(y_test, y_pred_svm_roc,multi_class='ovr')

auc_dts = metrics.roc_auc_score(y_test, y_pred_dts_proba,multi_class='ovr')

auc_rf = metrics.roc_auc_score(y_test, y_pred_rf_proba,multi_class='ovr')



plt.style.use('seaborn')

plt.plot(fpr_logistic, tpr_logistic, linestyle='--',color='purple', label='Logistic
Regression,auc='+str(auc_logestic))

plt.plot(fpr_knn,tpr_knn, linestyle='--',color='green', label='KNN,auc='+str(auc_knn))

plt.plot(fpr_svm,tpr_svm, linestyle='--',color='red', label='svm,auc='+str(auc_svm))
```

```
plt.plot(fpr_dts, tpr_dts, linestyle='--',color='purple', label='DTS ,Auc='+str(auc_dts))
plt.plot(fpr_rf,tpr_rf, linestyle='--',color='green', label='RF,Auc='+str(auc_rf))


plt.plot(p_fpr, p_tpr, linestyle='--', color='blue')
plt.title('ROC curve',fontsize=20)
plt.xlabel('FalsePositive Rate',fontsize=20)
plt.ylabel('True Positive rate',fontsize=20)
plt.legend(bbox_to_anchor=(1.2,1.05 ),loc='best',fontsize=15)
plt.show()
df_important.columns.tolist()
#for more accuracy and optimize testing,i put the formula into a dataframe.
#if you want to give several data,plz run this cell and belower again.
hsa_mir_185=float(input('pls enter hsa.mir.185:'))
hsa_mir_21=float(input('pls enter hsa.mir.21:'))
hsa_mir_146b=float(input('pls enter hsa.mir.146b:'))
hsa_mir_4326=float(input('pls enter hsa.mir.4326:'))
hsa_mir_93=float(input('pls enter hsa.mir.93:'))


df_cancer_final=pd.DataFrame({'hsa.mir.185':[hsa_mir_185],
                'hsa.mir.21':[hsa_mir_21],
                'hsa.mir.146b':[hsa_mir_146b],
                'hsa.mir.4326':[hsa_mir_4326],
                'hsa.mir.93':[hsa_mir_93],

                })
```

```
df3=df_important2.append(df_cancer_final)
df3.reset_index(drop=True,inplace=True)
df3
train=df3.iloc[:409]
test=df3.iloc[409:]
x_train=df3[['hsa.mir.185',
 'hsa.mir.21',
 'hsa.mir.146b',
 'hsa.mir.4326',
 'hsa.mir.93']][:409]
y_train=df_important[['target']][:409].values.reshape(-1,1)
x_test=df3[['hsa.mir.185',
 'hsa.mir.21',
 'hsa.mir.146b',
 'hsa.mir.4326',
 'hsa.mir.93']][409:]
model_svm.fit(x_train, y_train.ravel())
y_pred_svm=model_svm.predict(x_test)
if y_pred_svm[1]==0:
    print('normal')
else:
    print('cancer')
```
